# Supplementary material for: TCF Plus Radiochemotherapy Versus Neoadjuvant Radiochemotherapy Versus Flot Perioperative Chemotherapy in Esophageal Adenocarcinoma: The Results of a Three-Cohort, Multi-Centric Comparison: The A4 Study
Source: Biomedicines. 2025 Sep 11;13(9):2236. doi: 10.3390/biomedicines13092236 (PMC12467824; doi:10.3390/biomedicines13092236)
Supplement: Supplementary file 1 [file biomedicines-13-02236-s001.zip › biomedicines-3748374-Table S1.pdf]

Supplementary table 1. Distribution of haematological toxicity within the three cohorts.

Cohort	G0	G1	G2	G3	G4	G5	tot
A (RTCHT)	18	30	14	1	0	0	63
B (TCF plus RTCHT)	12	14	8	2	0	0	36
C (FLOT)	4	8	13	15	3	0	43
