# Supplementary material for: TCF Plus Radiochemotherapy Versus Neoadjuvant Radiochemotherapy Versus Flot Perioperative Chemotherapy in Esophageal Adenocarcinoma: The Results of a Three-Cohort, Multi-Centric Comparison: The A4 Study
Source: Biomedicines. 2025 Sep 11;13(9):2236. doi: 10.3390/biomedicines13092236 (PMC12467824; doi:10.3390/biomedicines13092236)
Supplement: Supplementary file 1 [file biomedicines-13-02236-s001.zip › biomedicines-3748374-Table S2.pdf]

Supplementary table 2. Distribution of preoperative and postoperative G≥2 toxicity within cohorts

**PREOPERATIVE TOXICITY**	**Cohort A**	**Cohort B**	**Cohort C**
Emesis and nausea	5	6	15
esophagitis	10	20	5
Radiation pneumonia	2	1	0
Fatigue	10	22	19
other	1	1	1
**POSTOPERATIVE TOXICITY**	**Cohort A**	**Cohort B**	**Cohort C**
Sepsis	8	2	5
Arithmia	5	4	4
Pulmonary complications (pleural effusion/pneumonia)	10	5	4
Anastomotic leakage	8	2	3
other	6	3	6
