# Supplementary figures and images for: TCF Plus Radiochemotherapy Versus Neoadjuvant Radiochemotherapy Versus Flot Perioperative Chemotherapy in Esophageal Adenocarcinoma: The Results of a Three-Cohort, Multi-Centric Comparison: The A4 Study
Source: Biomedicines. 2025 Sep 11;13(9):2236. doi: 10.3390/biomedicines13092236 (PMC12467824; doi:10.3390/biomedicines13092236)

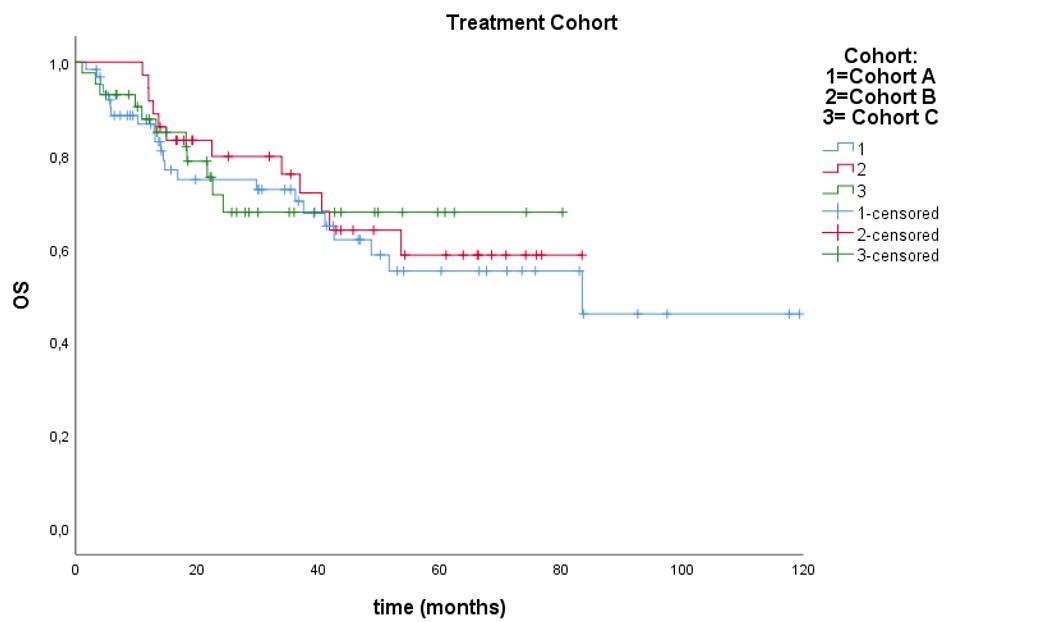

Supplement: Supplementary file 1 [file biomedicines-13-02236-s001.zip › biomedicines-3748374-Figure S1.jpg]
